# Supplementary material for: Translation and Validation of the Arabic Version of the Athlete Sleep Screening Questionnaire
Source: Healthcare (Basel). 2023 May 22;11(10):1501. doi: 10.3390/healthcare11101501 (PMC10218295; doi:10.3390/healthcare11101501)
Supplement: Supplementary file 1 [file healthcare-11-01501-s001.zip › healthcare-2228960-supplementary.pdf]

Athlete Sleep Screening Questionnaire (ASSQ)

INSTRUCTIONS

The following questions relate to your sleep habits. Please circle the best answer which you think represents your typical sleep habits over the recent past. For all questions, circle a letter from 'a' to 'e' unless otherwise specified.

1. During the recent past, how many hours of actual sleep did you get at night? (This may be different than the number of hours you spent in bed.)

a. 5 to 6 hours
b. 6 to 7 hours
c. 7 to 8 hours
d. 8 to 9 hours
e. more than 9 hours

2. How many naps per week do you take?

a. none
b. once or twice
c. three or four times
d. five to seven times

3. How satisfied/dissatisfied are you with the quality of your sleep?

a. very satisfied
b. somewhat satisfied
c. neither satisfied nor dissatisfied
d. somewhat dissatisfied
e. very dissatisfied

4. During the recent past, how long has it usually taken you to fall asleep each night?

a. 15 minutes or less
b. 16 – 30 minutes
c. 31 – 60 minutes
d. longer than 60 minutes

5. How often do you have trouble staying asleep?

a. none
b. once or twice per week
c. three or four times per week
d. five to seven days per week

6. During the recent past, how often have you taken medicine to help you sleep (prescribed or over-the-counter)?

a. none
b. once or twice per week

استبانة فحص نوم الرياضيين

التعليمات

تتعلق الأسئلة التالية بعادات نومك. يرجى وضع دائرة حول أفضل إجابة تعتقد أنها تمثل عادات نومك المعتادة في الماضي القريب .

١ .خلال الماضي القريب ، كم عدد ساعات نومك الفعلي في الليل؟ (قد يختلف هذا عن عدد الساعات التي قضيتها في السرير).

أ. من ٥ إلى ٦ ساعات
ب. من ٦ إلى ٧ ساعات
ج. ٧ إلى ٨ ساعات
د. من ٨ إلى ٩ ساعات
هـ. أكثر من ٩ ساعات

٢ .كم قيلولة تأخذها في الأسبوع؟

أ. ولا مره
ب. مره الي مرتين
ج. ثلاث الي اربع مرات
د. خمسه الى سبع مرات

٣ .ما مدى رضاك / عدم رضاك عن جودة نومك؟

أ. راض تماما
ب. راض إلى حد ما
ج. لست راضيًا ولا غير راضٍ
د. غير راض بعض الشيء
هـ. غير راض تماما

٤ .خلال الماضي القريب ، ما هي المدة التي استغرقتها عادة لتدخل في النوم كل ليلة؟

أ. ١٥ دقيقة أو أقل
ب. ١٦ - ٣٠ دقيقة
ج. ٣١-٦٠ دقيقة
د. أكثر من ٦٠ دقيقة

٥ .كم مرة تجد صعوبة في البقاء نائمًا؟

أ. ولا مرة
ب. مرة أو مرتين في الأسبوع
ج. ثلاث أو أربع مرات في الأسبوع
د. خمسة إلى سبعة أيام في الأسبوع

٦ .في الماضي القريب ، كم مرة تناولت دواءً لمساعدتك على النوم (بوصفة طبية أو بدون وصفة طبية)؟

أ. ولا مرة
ب. مرة أو مرتين في الأسبوع
ج. ثلاث أو أربع مرات في الأسبوع
د. خمس إلى سبع مرات في الأسبوع

٧ .ضع في اعتبارك "الشعور الأفضل" الخاص بك ، في أي وقت ستستيقظ إذا كان لك الخيار تمامًا للتخطيط ليومك؟

أ. ٥:٠٠ صباحًا – ٦:٣٠ صباحًا
ب. ٦:٣٠ صباحًا – ٧:٤٥ صباحًا
ج. ٧:٤٥ صباحًا - ٩:٤٥ صباحًا
د. ٩:٤٥ صباحًا - ١١:٠٠ صباحًا
هـ. ١١:٠٠ صباحًا - ١٢:٠٠ ظهرًا

c. three or four times per week
d. five to seven times per week

7. Considering only your own "feeling best" rhythm, at what time would you get up if you were entirely free to plan your day?
a. 5:00 am – 6:30 am
b. 6:30 am – 7:45 am
c. 7:45 am – 9:45 am
d. 9:45 am – 11:00 am
e. 11:00 am – 12:00 pm (noon)

8. How alert do you feel during the first half-hour after having awakened?
a. not at all alert
b. slightly alert
c. fairly alert
d. very alert

9. Do you consider yourself to be a morning type person or an evening type person?
a. definitely a morning type
b. more a morning type than an evening type
c. more an evening type than a morning type
d. definitely an evening type

10. **Considering your own "feeling best" rhythm, at what time would you go to bed if you were entirely free to plan your evening?**
a. 8:00 pm – 9:00 pm
b. 9:00 pm – 10:15 pm
c. 10:15 pm – 12:30 am
d. 12:30 am – 1:45 am
e. 1:45 am – 3:00 am

11. **When you are travelling for your sport, do you experience sleep disturbance?**
a. Yes
b. No

12. **When you are travelling for your sport, do you experience daytime dysfunction (feeling generally unwell or having poor performance)?**
a. Yes
b. No

13. Are you typically a loud snorer?
a. Yes
b. No

14. Have you been told that you choke,

٨ . .إلى أي مدى تشعر بالتنبه خلال أول نصف ساعة بعد الاستيقاظ؟
أ. ليس في حالة تنبه على الإطلاق
ب. تنبه طفيف
ج. إلى حد ما في حالة تنبه
د. في حالة تنبه شديد

٩ .هل تعتبر نفسك من النوع الصباحي أو المسائي؟
أ. بالتأكيد صباحي
ب. صباحي أكثر من مسائي
ج. مسائي أكثر من صباحي
د. بالتأكيد مسائي

١٠ . .ضع في اعتبارك "الشعور الأفضل" الخاص بك، في أي وقت تذهب إلى الفراش إذا كان لك الخيار تمامًا في التخطيط لأمسيتك؟
أ. ٨:٠٠ مساءً - ٩:٠٠ مساءً
ب. ٩:٠٠ م - ١٠:١٥ م
ج. ١٠:١٥ مساءً - ١٢:٣٠ صباحًا
د. ١٢:٣٠ صباحًا - ١:٤٥ صباحًا
هـ. ١:٤٥ صباحًا - ٣:٠٠ صباحًا

١١ .عندما تسافر لتمارس رياضتك ، هل تعاني من اضطرابات في النوم؟
أ. نعم
ب. لا
١٢ .عندما تسافر لتمارس رياضتك، هل تعاني من اختلال وظيفي أثناء النهار (شعور عام بالتوعك أو ضعف الأداء)؟
أ. نعم
ب. لا

١٣ .هل عادة ما تشخر بصوت عال؟
أ. نعم
ب. لا

١٤ .هل قيل لك أنك تختنق أو تلهث أو تتوقف عن التنفس لفترات من الوقت أثناء النوم؟
أ. نعم
ب. لا

١٥ .في المتوسط ، كم عدد منتجات الكافيين التي تتناولها يوميًا (مثل حبوب الكافيين ، أو القهوة ، أو الشاي ، أو الصودا ، أو مشروبات الطاقة) ؟
للقهوة والشاي: مشروب واحد =٦-٨ أوقية / ١٧٧-٢٣٧ مل ؛ بالنسبة للصودا المحتوية على الكافيين: مشروب واحد = علبة واحدة (١٢ أوقية / ٣٥٥ مل)
أ. أقل من ١ في اليوم
ب. ١-٢ في اليوم
ج. ٣ في اليوم
د. ٤ في اليوم
هـ. ٥ أو أكثر في اليوم

١٦ .في الماضي القريب، كم مرة تستخدم جهازًا إلكترونيًا (على سبيل المثال: هاتف جوال ، كمبيوتر ، جهاز لوحي/تابلت ، تلفزيون ، إلخ) خلال ساعة واحدة من الذهاب إلى الفراش؟

**gasp, or stop breathing for periods of time during sleep?**<br>a. Yes<br>b. No<br><br>**15. On average, how many caffeinated products (caffeine pills, coffee, tea, soda, energy drinks) do you have per day? For coffee and tea, one drink = 6-8oz/177-237ml; for caffeinated soda, one drink = 1 can (12oz/355ml)?**<br>a. Less than 1 per day<br>b. 1-2 per day<br>c. 3 per day<br>d. 4 per day<br>e. 5 or more per day<br><br>**16. Over the recent past, how often do you use an electronic device (example: cell phone, computer, tablet, T.V. etc.) within 1 hour of going to bed?**<br>a. Not at all<br>b. 1-3 times per week<br>c. 4-6 times per week<br>d. Every day	أ. ولا مرة على الإطلاق<br>ب. ١-٣ مرات في الأسبوع<br>ج. ٤-٦ مرات في الأسبوع<br>د. كل يوم
